# AKT phosphorylates H3-threonine 45 to facilitate termination of gene transcription in response to DNA damage

**DOI:** 10.1093/nar/gkv176

**Published:** 2015-03-26

**Authors:** Jong-Hyuk Lee, Byung-Hee Kang, Hyonchol Jang, Tae Wan Kim, Jinmi Choi, Sojung Kwak, Jungwon Han, Eun-Jung Cho, Hong-Duk Youn

**Affiliations:** 1 National Creative Research Center for Epigenome Reprogramming Network, Department of Biomedical Sciences, Ischemic/Hypoxic Disease Institute, Seoul National University College of Medicine, Seoul 110–799, Republic of Korea; 2 Division of Cancer Biology, Research Institute, National Cancer Center, Goyang 410-769, Republic of Korea; 3 Cancer Research Institute, Seoul National University College of Medicine, Seoul 110-799, Republic of Korea; 4 College of Pharmacy, Sungkyunkwan University, Suwon 440-746, Republic of Korea; 5 Department of Molecular Medicine and Biopharmaceutical Sciences, Graduate School of Convergence and Technology, Seoul National University, Seoul 110-799, Republic of Korea

## Abstract

Post-translational modifications of core histones affect various cellular processes, primarily through transcription. However, their relationship with the termination of transcription has remained largely unknown. In this study, we show that DNA damage-activated AKT phosphorylates threonine 45 of core histone H3 (H3-T45). By genome-wide chromatin immunoprecipitation sequencing (ChIP-seq) analysis, H3-T45 phosphorylation was distributed throughout DNA damage-responsive gene loci, particularly immediately after the transcription termination site. H3-T45 phosphorylation pattern showed close-resemblance to that of RNA polymerase II C-terminal domain (CTD) serine 2 phosphorylation, which establishes the transcription termination signal. AKT1 was more effective than AKT2 in phosphorylating H3-T45. Blocking H3-T45 phosphorylation by inhibiting AKT or through amino acid substitution limited RNA decay downstream of mRNA cleavage sites and decreased RNA polymerase II release from chromatin. Our findings suggest that AKT-mediated phosphorylation of H3-T45 regulates the processing of the 3′ end of DNA damage-activated genes to facilitate transcriptional termination.

## INTRODUCTION

N-terminal tails of core histones are rich in basic amino acids and undergo many post-translational modifications. These modifications correlate closely with transcription (1). DNA damage stimulates dynamic chromatin remodeling to induce various cellular responses to maintain genomic stability, which has been shown to be mediated through histone phosphorylation of key residues on histone N-terminal tails (2,3). For example, phosphorylation of S139 of the mammalian H2AX histone variant occurs in response to DNA damage by ataxia telangiectasia mutated (ATM) and ataxia telangiectasia and Rad3-related protein (ATR) (4,5). DNA double-strand break-induced phosphorylated H2AX, referred to as γH2AX, in turn, creates a platform to which DNA damage repair/chromatin structure modifier enzymes are recruited (6). In yeast, H4-S1 is phosphorylated by casein kinase II under genotoxic stress (7). H4 -S1 is phosphorylated in late stages of DNA damage repair and stabilizes the restored chromatin structure by counteracting H4 acetylation by NuA4 (8). Mammalian H2B-S14 is also phosphorylated by ionizing radiation and co-localizes with γH2AX foci (9).

Despite these insights, however, the link between DNA damage-induced histone phosphorylation and transcriptional regulation remains poorly understood. Post-translational modifications of histone proteins in chromatin are critical in gene transcription, regulating their access to transcriptional regulators by altering the electrostatic or structural characteristics of chromatin. Acetylated histones H3 and H4 are detected in the promoter regions of actively transcribed genes. H3-K4 trimethylation peaks during transcriptional initiation, whereas di- and trimethylated H3-K36 occupies these sites throughout transcriptional elongation (10).

Transcription in eukaryotes is mediated by RNA polymerase II and termination is a prerequisite for genomic partitioning and precise expression of neighboring genes (11). Particularly, 3′ end processing is an essential step in mRNA maturation, splicing and transcriptional termination (12). Two models have been proposed to explain 3′ end processing and RNA pol II transcriptional termination. The ‘allosteric’ or ‘anti-terminator’ model (13) suggests that when RNA pol II-mediate transcription progresses through the poly(A) site, the elongation complex undergoes a conformational change that leads to the dissociation of elongation factors and the recruitment of termination factors. In the ‘torpedo’ model (14), mRNA cleavage at the poly(A) site generates unprotected downstream RNA products, which are attacked by 5′-3′ exonucleases. The exonuclease (torpedo) catches up to RNA pol II and releases it from chromatin. Emerging evidence indicates that the transcriptional termination mechanism is likely to be a sophisticated combination of both models (15). Despite its importance, termination is arguably the least understood step in transcription. Furthermore, the relationship between transcriptional termination and histone modifications has remained largely unknown.

In this study, we demonstrate that also known as protein kinase B (AKT) phosphorylates threonine 45 of histone H3 (H3-T45) upon DNA damage and that AKT-mediated phosphorylation of H3-T45 regulates transcriptional termination. These findings unravel an unexpected function of AKT—as a histone kinase—in the transcriptional termination of genes in response to DNA damage.

## MATERIALS AND METHODS

### Cell culture and transient expression

HeLa, MCF7 and MCF10A cells were obtained from American Type Culture Collection (ATCC). Except for MCF10A, the cells were cultured in Dulbecco's modified Eagle's medium, supplemented with 10% (v/v) fetal bovine serum (FBS) and antibiotics. MCF10A cells were cultured in DMEM-F12 media, supplemented with 5% (v/v) FBS, L-glutamine, antibiotics, insulin (20 μg/ml, Sigma), cholera toxin (0.1 μg/ml, Listbiological Labs), hydrocortisone (1 μg/ml, Sigma) and hEGF (0.02 μg/ml, PeproTech). Polyethylenimine (PEI, Polysciences) was used to transfect HeLa cells. MCF10A cells were electrophoresed with the Neon Transfection System (Invitrogen) per the manufacturer's instructions.

### DNA constructs and purification of recombinant proteins

Mammalian expression vectors for dominant negative (DN) and myristoylated (Myr) AKT1 were purchased from Millipore. Expression vectors for wild-type (WT) and T45A H3 were generated by subcloning the corresponding polymerase chain reaction (PCR) fragment into the Platinum-A (Plat-A) cell line and MCF10A cells that stably overexpressed these clones were generated per the manufacturer's instructions (CELL BIOLAB, INC.). Core histone proteins were generated by subcloning the corresponding PCR fragment into pGEX4T-1 (Amersham Biosciences) or pRSETB (Invitrogen). glutathione S-transferase (GST) fusion proteins were expressed in the *Escherichia coli* strain *DH5α* and proteins were isolated using Glutathione Sepharose™ 4B beads (Amersham Biosciences) per the manufacturer's instructions. His_6_ fusion proteins were expressed in the *BL21DE*3 strain and purified with Ni-NTA agarose (Qiagen) per the manufacturer's instructions.

### Antibodies and reagents

Anti-actin was purchased from Sigma; anti-Myc (9E10) was obtained from Covance; anti-pan H3, anti-H3 (phosphorylated-T45, trimethylated-K36) were purchased from Abcam; anti-H2A, anti-H2B, anti-H3 (phosphorylated-S10), anti-H2A/H4 (phosphorylated-S1) and anti-H2AX (phosphorylated-S139) were acquired from Upstate; anti-H2B (phosphorylated-S36) was obtained from ECM Biosciences; anti-phosphorylated RNA Pol II-S2 (3E10) and anti-phosphorylated RNA Pol II-S5 (3E8) were purchased from Millipore; anti-p53 (DO-1, FL393) and anti-Rpb1 were purchased from Santa Cruz; and anti-AKT1, anti-AKT2, anti-pan AKT and anti-AKT (phosphorylated-S473) were acquired from Cell Signaling.

Rabbit polyclonal H3-T45 phosphorylation-specific antibody was generated from Openbiosystems (Thermofisher Scientific) by immunizing three Specific Pathogen Free (SPF) rabbits with phosphorylated H3-T45 peptide. H3 antibodies (phosphorylated-T3, T11, S28) were a generous gift from Dr Kyong-Tai Kim (Department of Life Science, Division of Molecular & Life Science, Pohang University of Science and Technology).

### Lentiviral sh-RNA-mediated knockdown of AKT

Lentiviral vectors that contained the human AKT1-targeting sequences pLKO.1-sh-AKT1 #1 (TRCN0000010171), #2 (TRCN0000010174), #3 (TRCN0000039794), #4 (TRCN0000039795), and #5 (TRCN0000039793) and AKT2 pLKO.1-sh-AKT2 #1 (TRCN0000000563), #2 (TRCN0000039968), #3 (TRCN0000265834), #4 (TRCN0000265848) and #5 (TRCN0000265851) were purchased from Sigma. As a control, the pLKO.1 vector was used. Lentivirus was produced per the manufacturer's protocol using the BLOCK-iT Lentiviral RNAi expression system (Invitrogen). Twenty-four hours after lentiviral infection, infected cells were selected with puromycin (1 μg/ml) for 2 weeks and then used in the experiments. Because pLKO.1-sh-AKT1 #3 and pLKO.1-sh-AKT2 #2 were the most effective, we used these vectors in most experiments, unless specifically noted.

### AKT1 kinase assay

Anti-Myc conjugated protein G agarose beads were used to immunoprecipitate total lysates of HEK293T cells that overexpressed DN/Myr-AKT1. Immunoprecipitated protein G agarose beads or active AKT1 (Upstate) was mixed with substrate in 20 mM MOPS (pH 7.2), 25 mM β-glycerol phosphate, 5 mM EGTA, 1 mM sodium orthovanadate, 1 mM dithiothreitol, 75 mM MgCl_2_, 75 μM ATP and 10 μCi ^32^P-ATP. The reaction mixture was incubated for 2 h at 30°C and loaded onto sodium dodecyl sulphate-polyacrylamide gel electrophoresis gels. The gels were dried for 1 h at 80°C and exposed to X-ray film overnight at −80°C.

### Immunofluorescent staining

Immunofluorescent staining was performed as described (16).

### Chromatin immunoprecipitation assay

MCF10A cells (4 × 10^7^) were harvested and crosslinked with formaldehyde to a final concentration of 1%. The crosslinking reaction was stopped by adding glycine to a final concentration of 0.125 M. The cells were harvested and washed twice with cold phosphate buffered saline and cytosolic fractions were eliminated with buffer A [5 mM PIPES (pH 8.0), 85 mM KCl, 0.5% NP-40, protease inhibitors]. Nuclear pellets were washed and resuspended in 1× micrococcal nuclease reaction buffer [10 mM Tris–Cl (pH 7.9), 5 mM CaCl_2_, 0.5 mM DTT] and the chromatin was digested with micrococcal nuclease (New England Biolabs). The digestion was stopped with EDTA. The remainder of the procedure was performed as described (17).

### ChIP sequencing

MCF10A cells were ChIPed with anti-phosphorylated H3-T45, anti-phosphorylated RNA Pol II-S2 and S5, and anti-H3-K36me3. Sequencing and analysis of ChIPed DNA were performed by BML (Bio Medical Laboratories, Korea) and KRIBB (Korean Research Institute of Bioscience & Biotechnology). Briefly, DNA fragments were ligated to a pair of adaptors for sequencing on an Illumina Hiseq-2000. The ligation products were size-fractionated to obtain 200–300-bp fragments on a 2% agarose gel and PCR-amplified for 18 cycles. Each library was diluted to 8 pM for 76 cycles of single-read sequencing on the Illumina Hiseq-2000 following the manufacturer's recommended protocol.

### ChIP-sequencing data analysis

The sequencing reads were mapped against the human genome (GRCh37/hg19) using Bowtie v.2.1.0 (18) with default parameters. The SAM format outputs were sorted by genomic coordinates and uniquely mapped reliable reads were used in subsequent steps. SAM files were preprocessed using Picard (http://picard.sourceforge.net). We used the MACS v.1.4.2 tool (19) to select regions that were enriched for RNA Pol II and histone modifications. We applied the default settings and found significant regions (*P*-value ≤ 10 – 5) compared with matched control samples. Using HOMER (20), H3-T45 phosphorylation peaks were annotated for promoters, exons, introns, intergenic regions and other features, based on RefSeq transcripts. GO annotation was performed using GREATv2.0.2 (21). The average normalized tag count distribution around coding gene locations was plotted using ngs.plot (22).

### Nascent RNA quantification

Nascent RNA quantification was performed using the Nascent RNA capture kit (Life Technologies) per the manufacturer's recommendations. Briefly, 0.1 mM 5-ethynyluridine (EU) was added to the cell culture medium for 18 h and total RNA was extracted with TriZol^®^ (Invitrogen). Five micrograms of total RNA was biotinylated with reaction cocktail (50% v/v 2X EU buffer, 2 mM CuSO_4_, 0.5 mM biotin azide, 10 mM reaction additive 1, 12 mM reaction buffer additive 2) and vortexed for 30 min at room temperature. RNA was precipitated with glycogen, ammonium acetate and 100% ethanol at −80°C overnight. One microgram of biotinylated RNA was purified with streptavidin-coated magnetic beads, washed and reverse-transcribed using Superscript III reverse-transcriptase (Invitrogen) per the manufacturer's protocol. cDNA was treated with RNaseA (Invitrogen) at 37°C for 30 min and used as template for real-time qPCR analysis.

### Quantitative real-time PCR (qPCR) and conventional PCR analysis of relative mRNA levels and ChIP products

Total RNA was extracted with TriZol^®^ (Invitrogen) and reverse-transcribed (AMV Reverse Transcriptase XL, Life Science, Co. for real-time PCR analysis; Superscript III, Invitrogen, for conventional PCR analysis). mRNA and antibody-bound chromatin levels by chromatin immunoprecipitation assay were quantified by real-time qPCR with the SYBR^®^ Green qPCR Kit (Finnzymes, F-410L) on the iQ5 and CFX Connect Real-time PCR Detection System (Bio-Rad) and then normalized to actin or 1% input chromatin using the 2^−ΔΔ^*C*_T_2-ΔΔCT calculation method. Conventional PCR was performed using EX Taq polymerase (Takara) on a MyCycler (Bio-Rad). The sequences of the primers for mRNA quantification are listed in Supplementary Table S1.

### Statistics

Data are presented as means ± standard deviations and *P*-values were calculated using the student's *t*-test calculator (http://www.physics.csbsju.edu/stats/t-test.html). A value of *P* < 0.05 was considered to be statistically significant. All data are representative of at least three independent experiments.

## RESULTS

### AKT phosphorylates H3-T45 in response to DNA damage

We attempted to identify the phosphorylation sites on core histones in response to DNA damage (Supplementary Figure S1). Thus, MCF10A normal breast epithelial cells were treated with adriamycin and key phosphorylation sites were examined by western blot. As expected, we observed the induction of phosphorylated H2AX-S139 (4,5). Based on the decrease in phosphorylation of the mitotic-specific marker H3-T3, S10 and S28 (2), we anticipated that adriamycin-treated MCF10A cells would be undergoing severe cell cycle arrest, which was confirmed by the decline in H3-T11 phosphorylation.

At the promoter region, DNA damage quickly reduced Chk1-mediated phosphorylation of H3 -T11, repressing the transcription of cell cycle-activating genes such as cyclin B1 (23). Phosphorylation of transcription repression/activation-associated H2A/H4-S1 (8,24) was unaffected by adriamycin and phosphorylation of metabolic stress-associated H2B-S36 rose slightly (25). However, H3-T45 phosphorylation was greatly increased upon DNA damage. By confocal microscopy, we found that the fold-induction of H3-T45 phosphorylation did not exceed that of γH2AX. Unlike H2AX phosphorylation, H3-T45 was phosphorylated at low level, even under normal conditions. In contrast to γH2AX, which formed speckled foci on DNA double-strand break sites, H3-T45 phosphorylation was broader and had a nuclear-wide distribution (Supplementary Figure S2).

**Figure 1.**
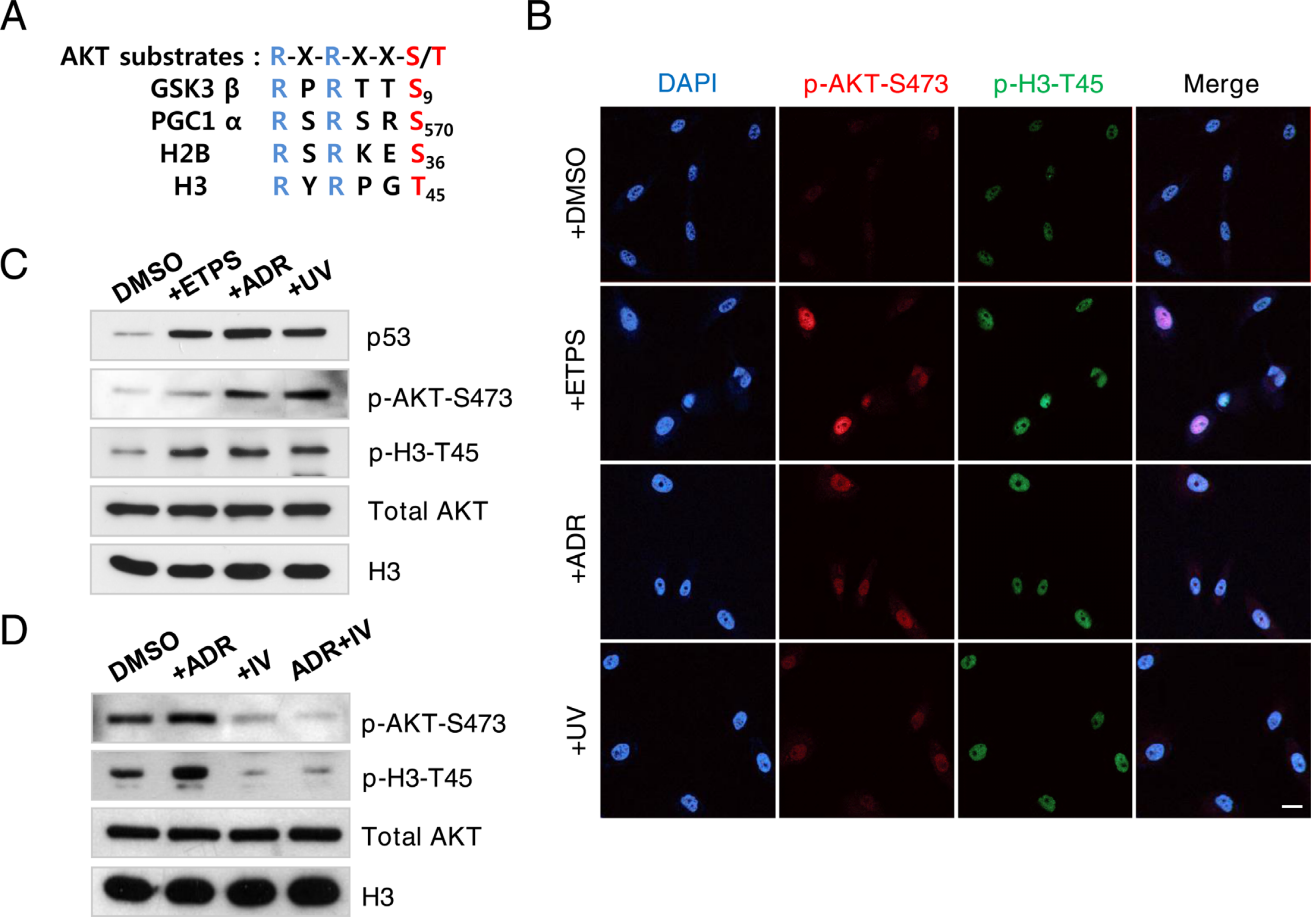
AKT phosphorylates H3–45 in response to DNA damage. (**A**) AKT substrate sequences conserved in various proteins, including histones H2B and H3. (**B**) Immunofluorescence staining of phosphorylated AKT-serine 473 (p-AKT-S473) and phosphorylated H3-threonine 45 (p-H3-T45) in MCF10A cells. Cells were treated with DMSO, 100 μM etoposide (ETPS), 0.4 μg/ml adriamycin (ADR) or 50 J/m^2^ UV irradiation (UV) for 18 h. DNA counterstained with DAPI; scale bar, 20 μm. (**C**) Western blot of samples in (B). (**D**) MCF10A cells were treated with DMSO, 0.4 μg/ml ADR, 0.2 μM AKT inhibitor IV (IV) or 0.4 μg/ml ADR and 0.2 μM AKT inhibitor IV for 18 h. Total cell extracts were probed by western blot. Data shown are the representative of three independent experiments.

By computational analysis (http://scansite.mit.edu), AKT was predicted to be the H3-T45 kinase with highest stringency and the amino acid sequence of H3-T45 harbors a perfectly conserved substrate recognition motif for AKT (Figure 1A). Moreover, AKT is activated by mitogenic growth factors and DNA damage (26), prompting us to test whether H3-T45 is phosphorylated by AKT. In MCF10A cells, the phosphorylation of AKT and H3-T45 increased with etoposide (ETPS), adriamycin (ADR) and UV exposure (Figure 1B and C). AKT inhibitor IV is a cell-permeable drug that targets an ATP-binding site of a kinase that lies immediately upstream of AKT but downstream of PI3K, specifically inhibiting AKT phosphorylation (27). The enhanced phosphorylation of AKT and H3-T45 with ADR treatment was mitigated by AKT inhibitor IV (Figure 1D and Supplementary Figure S3), supporting the notion that AKT is likely the kinase that phosphorylated H3-T45.

### AKT phosphorylates H3-T45 *in vitro* and *in vivo*

DNA damage-induced histone phosphorylation is associated with the regulation of chromatin states (2–9). Thus, we examined whether this site was phosphorylated by AKT on DNA damage.

**Figure 2.**
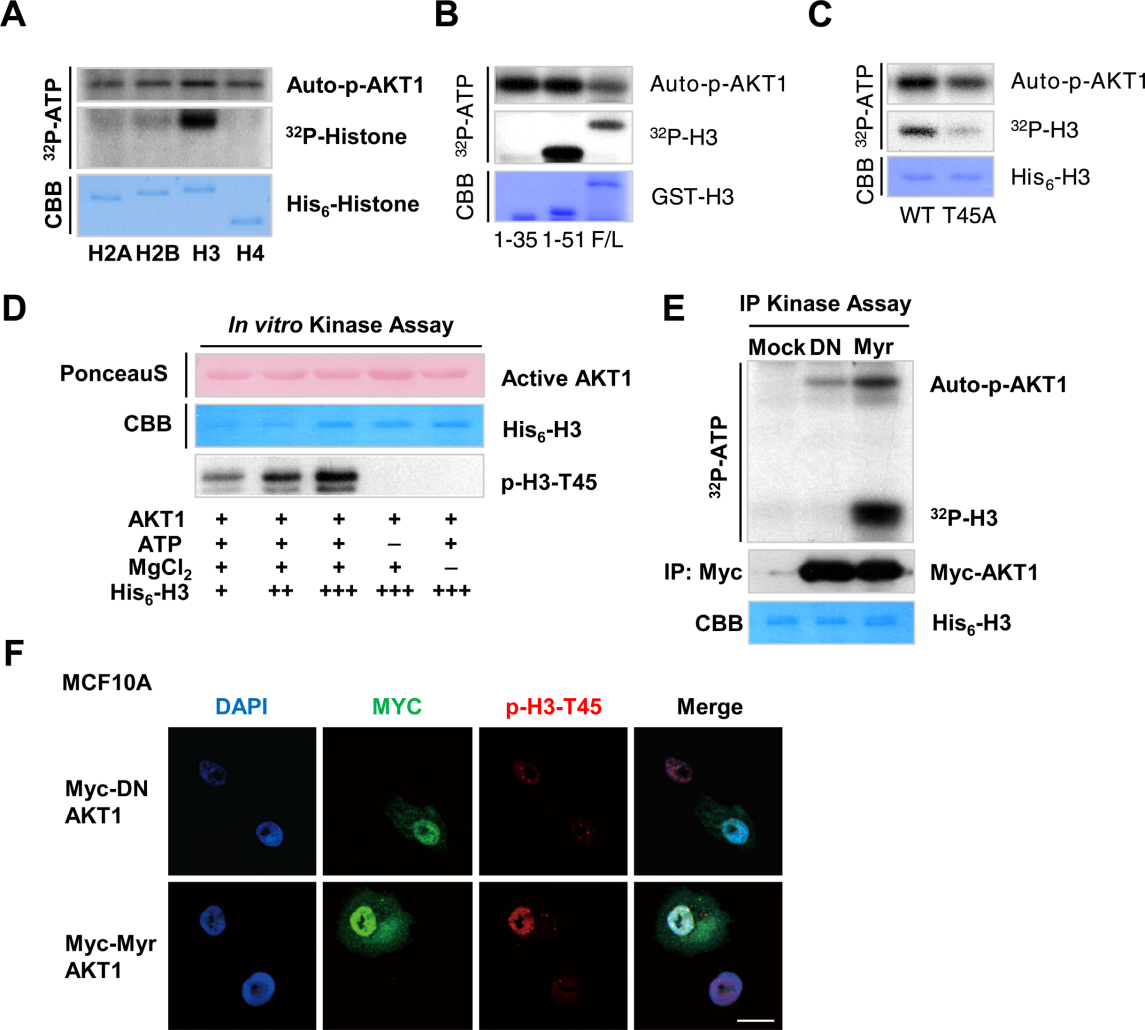
AKT1 phosphorylates H3-T45 *in vitro* and *in vivo*. (**A**) *In vitro* kinase assay of recombinant His_6_-tagged core histones purified from *Escherichia coli*. (**B**) AKT1 *in vitro* kinase assay of His_6_-H3. F/L indicates the full-length of H3. 1–35 and 1–51 represent the corresponding amino acid sequences of purified H3. (**C**) AKT1 *in vitro* kinase assay of full-length H3, wild-type (WT) and T45 mutated to alanine (T45A). Autophosphorylated AKT1 (auto-p-AKT1) is shown as a kinase loading control. (**D**) *In vitro* AKT1 kinase assay with/without non-radiolabeled ATP or MgCl_2_. Assay samples were probed with anti-phosphorylated H3-T45. (**E**) IP kinase assay of Myc-tagged blank vector and dominant-negative (DN) and constitutively-active myristoylated (Myr) AKT1. AKT1 constructs were transfected into HEK293T cells and cell lysates were immunoprecipitated with anti-Myc and subjected to *in vitro* kinase assay using His_6_-H3 as a substrate. (**F**) Immunofluorescence staining of Myc-tagged DN/Myr-AKT1 overexpressed in MCF10A cells. DNA counterstained with DAPI. Scale bar, 20 μm. All data above are the representative of three independent experiments.

Using the recombinant proteins (purified from *E. coli*), AKT was found to interact directly with histones and phosphorylated H3-T45 *in vitro* (Figure 2A–C, Supplementary Figure S4A and B). By *in vitr*o AKT kinase assay, we found that H3-T45 phosphorylation by AKT requires cofactors for phosphorylation (ATP, MgCl_2_) (Figure 2D). Further, by *in vitro* IP AKT kinase assay, the constitutively active (myristoylated; Myr) form of AKT1 phosphorylated histone H3, while the dominant-negative (DN) AKT1 failed to do so (Figure 2E and Supplementary Figure S4C). We confirmed that Myr-AKT1, but not DN-AKT1, phosphorylates H3-T45 in MCF10A cells by confocal microscopy, suggesting that H3-T45 is a direct substrate of AKT (Figure 2F and Supplementary Figure S6).

### H3-T45 phosphorylation signal is most abundant near the TTS

**Figure 3.**
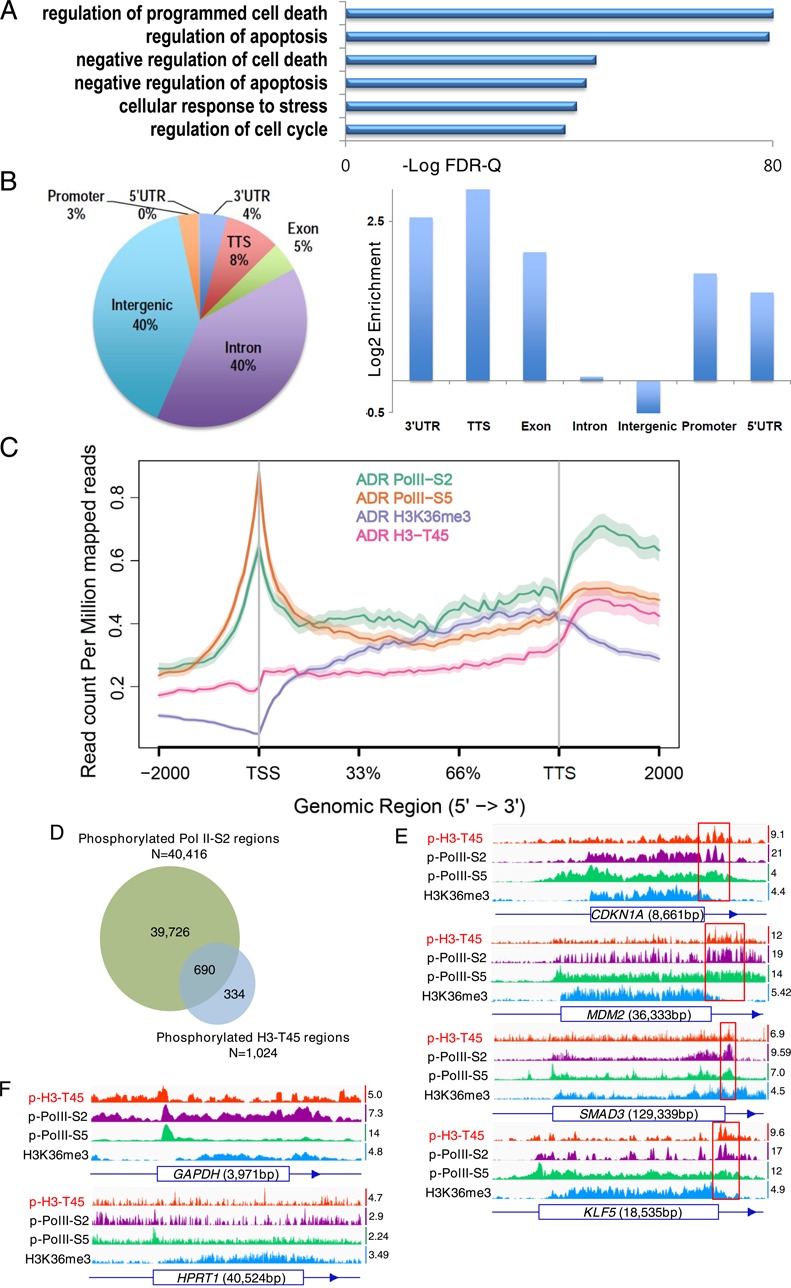

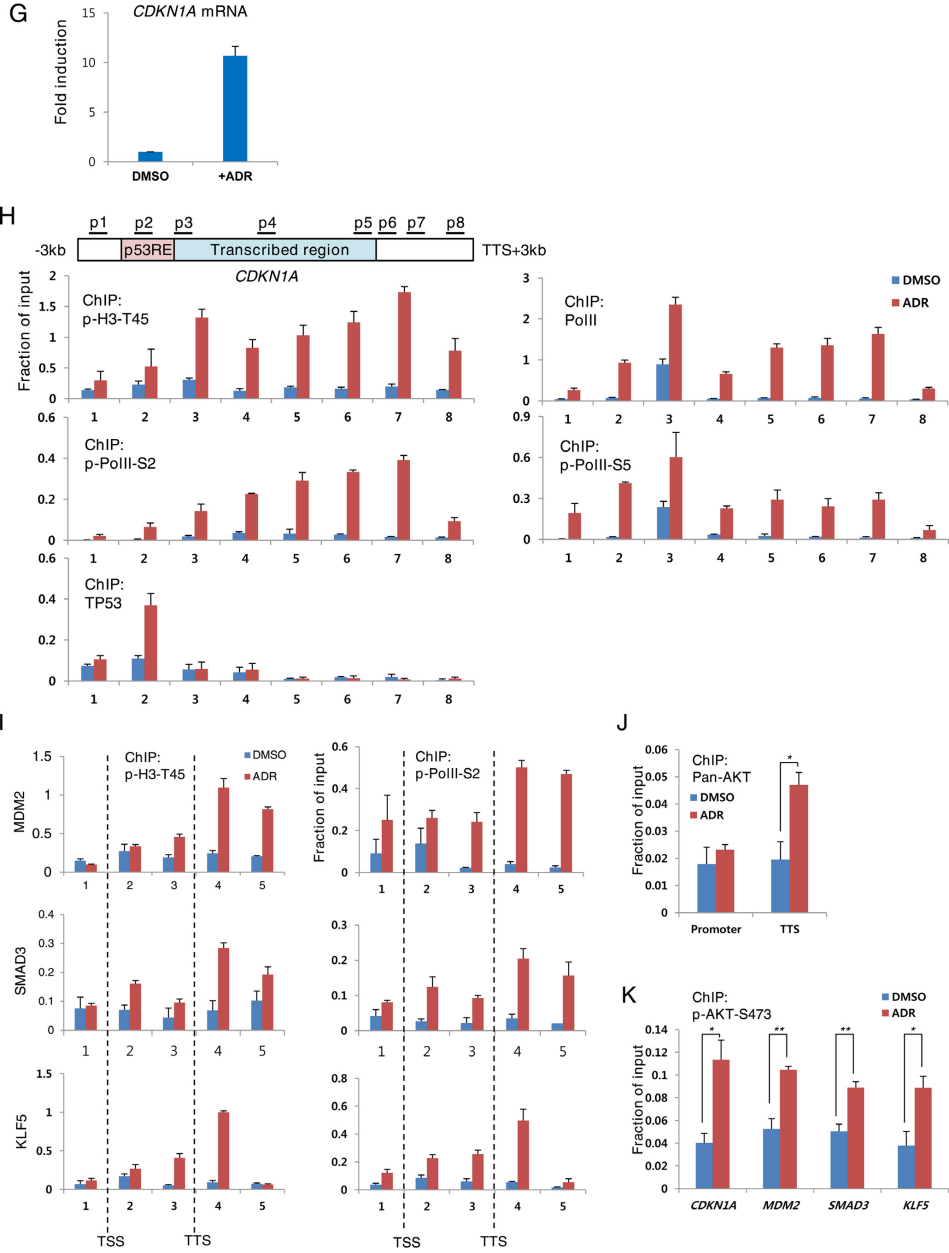
H3-T45 phosphorylation signal is most abundant near the TTS. MCF10A cells were treated with 0.4 μg/ml ADR for 18 h and analyzed by ChIP-seq. (**A**) Functional annotation of H3-T45 phosphorylation peak. (**B**) Pie chart showing the proportion of transcript coordinates for H3-T45 phosphorylation peaks (1041 regions). Proportion of transcript coordinates for H3-T45 phosphorylation peaks were compared with RefSeq transcripts. (**C**) Average profiles for phosphorylated Pol II-S2, S5, H3K36me3 and phosphorylated H3-T45 were plotted around ADR-induced H3-T45 phosphorylation-enriched genes (610 genes). (**D**) Phosphorylated RNA Pol II-S2 and phosphorylated H3-T45 ChIP peak distribution. (**E** and **F**) ChIP binding profiles of indicated genes. Scale data ranges are indicated on the right side of the individual track. Red boxes indicate the highest peak of phosphorylated H3-T45 signal. (**G**) Real-time qPCR analysis of *CDKN1*A mRNA in DMSO/ADR-treated MCF10A cells. (**H**) ChIP assay covering the *CDKN1A* locus above with the indicated antibodies. (**I**) ChIP-qPCR of the indicated gene locus with anti-phosphorylated H3-T45 and anti-phosphorylated RNA Pol II-S2. (**J**) ChIP-qPCR of promoter and TTS of *CDKN1A*, using anti-pan AKT. (**K**) ChIP-qPCR using anti-phosphorylated AKT-S473. qPCR was performed with complementary primers to the TTS of the indicated genes. ChIP-pPCR values were normalized with 1% input DNA. Real-time qPCR and ChIP assay data shown are the average values of at least three independent experiments. Standard deviations are indicated as error bars. * P < 0.05, **P < 0.001.

H3-T45 lies in the N-terminus of the first helix of H3 and constitutes the nucleosome entry/exit point. This residue is assumed to have significant function, because it makes contact with genomic DNA (28–30). MCF10A cells were treated with ADR, and genome-wide ChIP-seq was performed with phosphorylated H3-T45 antibody, yielding a set of genes that contained phosphorylated H3-T45 (Supplementary Table S2). In our functional annotation analysis, most phosphorylated H3-T45-positive regions lay in cellular stress-responsive genes (Figure 3A and Supplementary Figure S7); the signal appeared primarily in the 3′ UTR of the gene-coding region (Figure 3B, C and Supplementary Figure S8). The specific pattern of H3-T45 phosphorylation, which centered around the transcription termination site (TTS), resembled that of phosphorylated RNA polymerase II CTD-Ser2 (Pol II-S2)/transcription termination factors (11,31). In comparing ChIP-seq profiles (Supplementary Table S2), we noted that over 67% of H3-T45 phosphorylation overlapped with RNA Pol II-S2 phosphorylation (Figure 3D and E). Housekeeping genes, such as *GAPDH* and *HPRT1*, were phosphorylated H3-T45-negative (Figure 3F), with lower RNA Pol II-S2 phosphorylation levels than H3-T45-positive genes (Figure 3E and F; RNA Pol II-S2 scale data ranges are 21 and 19 on *CDKN1A* and *MDM2* versus 7.3 and 2.9 on *GAPDH* and *HPRT1*, respectively).

Inspection of the *CDKN1A* locus by ChIP-qPCR recapitulated the genome-wide ChIP-seq results. Phosphorylated H3-T45 signals in *CDKN1A* rose significantly on induction of *CDKN1A* transcription by ADR, peaking 3′ of the TTS, where RNA Pol II dissociated from the chromatin (Figure 3G and H). Consistent with the ChIP-seq data, the phosphorylation patterns of H3-T45 in *CDKN1A*, *MDM2*, *SMAD3* and *KLF5* were similar to that of RNA Pol II-S2 phosphorylation (Figure 3I). Moreover, the occupancy of AKT on the TTS of *CDKN1A* increased significantly upon adriamycin treatment, whereas that on the *CDKN1A* promoter was unaffected (Figure 3J and K).

CDC7, PKC-δ and DYRK1A have been reported to phosphorylate H3-T45 (28,30,32). Thus, we knocked down these kinases by lentiviral expression of sh-RNAs (Supplementary Figure S9A) and tested whether these kinases affected H4-T45 phosphorylation under DNA damaging conditions. Upon adriamycin treatment, knockdown of these kinases (PKC-δ, CDC7 and DYRK1A) did not alter H3-T45 phosphorylation (Supplementary Figure S9B). Moreover, H3-T45 phosphorylation at the TTS of *CDKN1A*, *MDM2*, *SMAD3* and *KLF5* did not change significantly by their knockdown (Supplementary Figure S9C), indicating that AKT, at least, is an H3-T45 kinase under DNA damage conditions. These data suggest that DNA damage-induced H3-T45 phosphorylation by AKT occurs exclusively 3′ of the TTS in actively transcribed genes, where RNA Pol II-S2 phosphorylation is maximized.

### AKT1 phosphorylates H3-T45 more efficiently than AKT2

**Figure 4.**
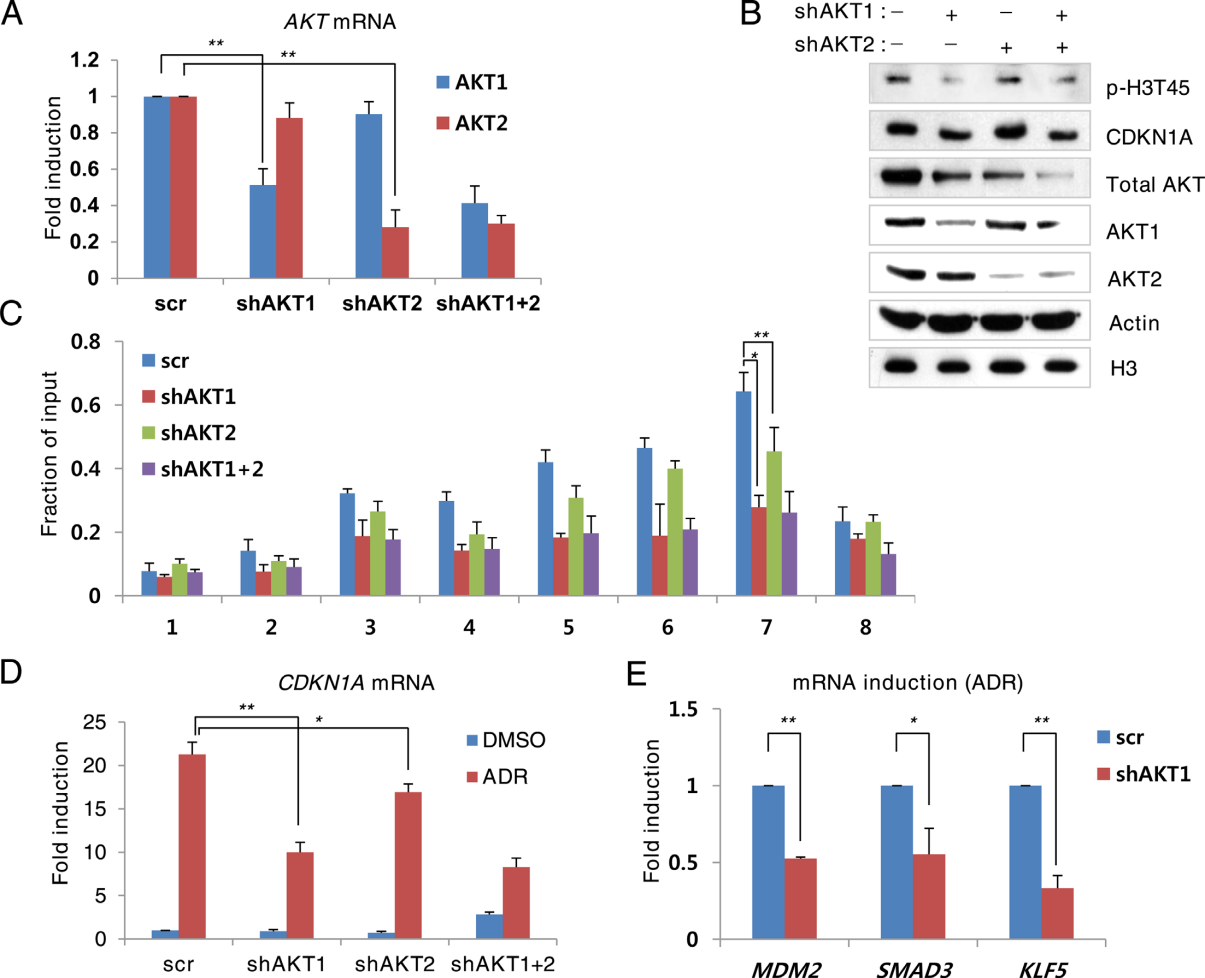
AKT1 phosphorylates H3-T45 phosphorylation more efficiently than AKT2. AKT knockdown MCF10A cells were treated with 0.4 μg/ml ADR for 18 h. (**A**) Real-time PCR analysis of *AKT* mRNA. (**B**) Western blot analysis of total cell extracts with the indicated antibodies. (**C**) Phosphorylated H3-T45 ChIP assay of the *CDKN1A* locus. (**D**) Real-time PCR analysis of *CDKN1A* mRNA. (**E**) Real-time qPCR analysis of the indicated genes in ADR-treated MCF10A cells. Real-time qPCR and ChIP assay data shown are the average values of at least three independent experiments. Standard deviations are indicated as error bars. *P < 0.05, **P < 0.001.

In human cells, *AKT1*, *AKT2* and *AKT3*, on chromosomes 14, 19 and 1, respectively, encode distinct AKT isoforms (33). AKT1 and AKT2 are ubiquitously expressed and AKT3 is tissue- and cell type-specific. To determine the AKT isoforms that mediate H3-T45 phosphorylation, we generated AKT1 and AKT2 knockdown MCF10A cells (Figure 4A and B). By ChIP-qPCR, knockdown of AKT1 suppressed H3-T45 phosphorylation by ADR (Figure 4C). The level of *CDKN1A* mRNA was consistent with the ChIP-qPCR results, showing significantly lower expression upon AKT1 knockdown (Figure 4D). In contrast, AKT2 knockdown only had a moderate effect on H3-T45 phosphorylation and *CDKN1A* transcription. Consistent with *CDKN1A* mRNA, *MDM2*, *SMAD3* and *KLF5* mRNA levels fell significantly by AKT1 knockdown (Figure 4E). Together, these results strongly suggest that AKT1 is the primary if not the only kinase that phosphorylates H3-T45 in response DNA damage.

### H3-T45 phosphorylation is critical for transcriptional termination

**Figure 5.**
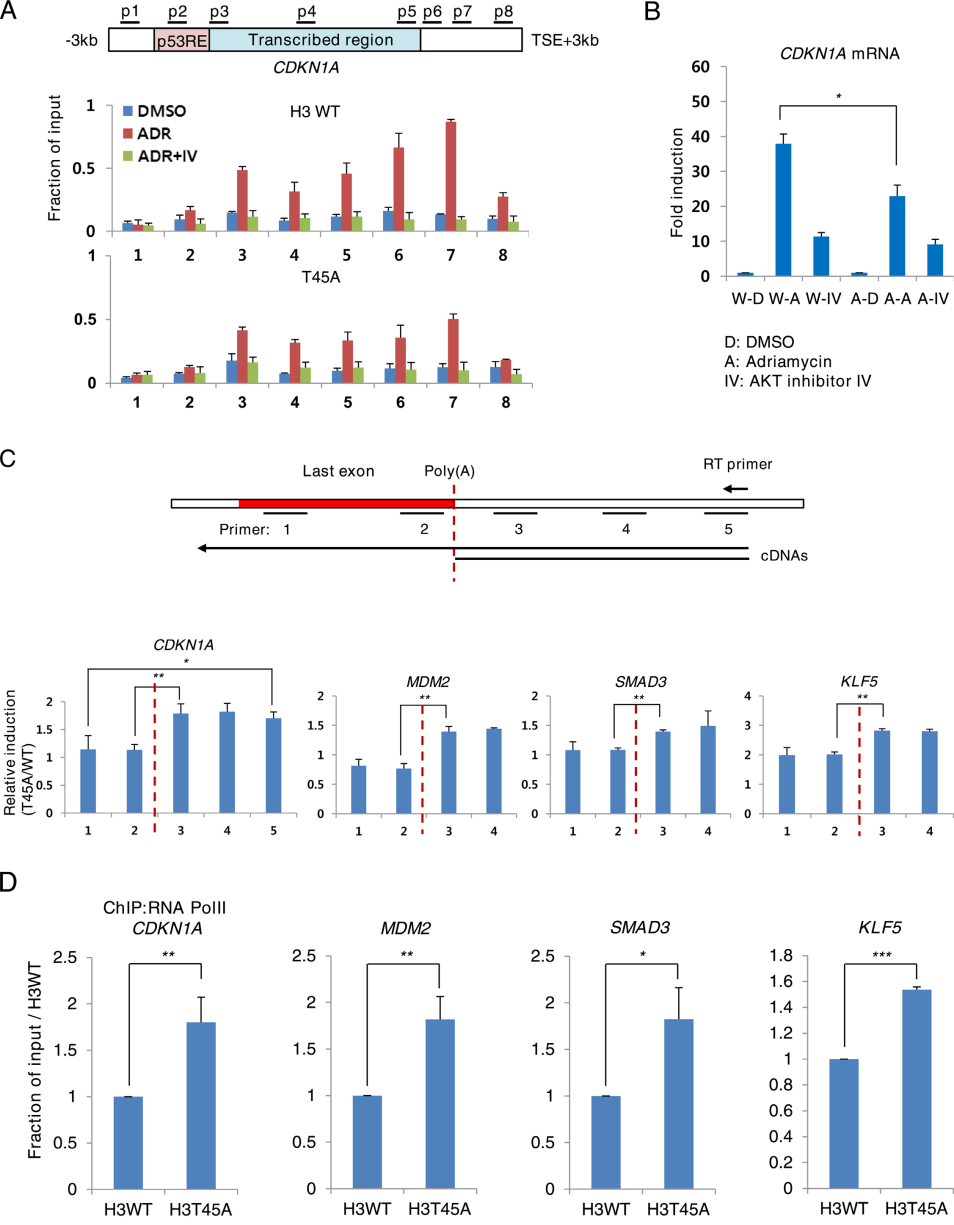
H3-T45 phosphorylation is critical for transcriptional termination. (**A**) ChIP assay of phosphorylated H3-T45 from H3 WT or T45A mutant overexpressing MCF10A cells. (**B**) Real-time qPCR of *CDKN1A* mRNA in cells from (A) prior to crosslinking. (**C**) Real-time qPCR analysis of nascent RNA of H3 WT or T45A mutant-overexpressing MCF10A cells, treated with ADR. Values represent the fold induction, relative to WT. (**D**) MCF10A cells expressing WT H3 and T45A mutant were treated with ADR for 18 h. ChIP assay was performed with anti-RNA Pol II. Real-time qPCR was performed with primers corresponding to the TTS of the indicated genes. Real-time qPCR and ChIP assay data shown are the average values of at least three independent experiments. Standard deviations are indicated as error bars. *P < 0.05 **P < 0. 01 ***P < 0.001.

To examine the effect of H3-T45 phosphorylation on transcriptional termination, we generated MCF10A cells that stably overexpressed a phosphorylation-defective mutant of H3-T45 (T45A) (Figure 5A). Despite the residual endogenous H3, the T45A mutation and AKT inhibitor IV suppressed ADR-induced *CDKN1A* transcription (Figure 5B). To determine whether AKT inhibitor IV treatment or the T45A mutation affected 3′-end processing, which is a crucial step in transcriptional termination (31), total RNA was reverse-transcribed with a complementary primer that encompassed the 3′ region of *CDKN1A*. This primer generates two complementary DNAs (cDNAs): a long, uncleaved pre-mRNA transcript and a shorter transcript that results from cleavage at the poly(A) site. The cDNA was analyzed with primer pairs to three regions: (i) the last intronic region, amplifying pre-mRNA (not mature mRNA), (ii) the poly(A) site, to detect uncleaved pre-mRNA product and (iii) the downstream RNA after cleavage at the poly(A) site, generating a product that lacks a 5′ cap and is degraded rapidly by exonucleases (34).

In DMSO- and ADR-treated cells, the amounts of PCR products that were generated up- and downstream of the poly(A) site were similar, despite the induction of pre-mRNA (Supplementary Figure S10). However, the levels of downstream product increased in AKT inhibitor-treated cells and T45A mutant cells. The amount of product that spanned the poly(A) site was unaltered by AKT inhibition or the T45A mutation, indicating that they affect the degradation of downstream RNA products after poly(A) site cleavage but not cleavage of the mRNA proper.

Next, we used a nascent RNA capture system, which quantifies transcripts precisely (35). With this method, EU is added to culture medium and incorporated into newly transcribed RNA, replacing uridine. Then, EU is biotinylated by copper-catalyzed cycloaddition and isolated by affinity separation. We observed an increase in 3′ downstream RNA products in *CDKN1A*, *MDM2*, *SMAD3* and *KLF5* (Figure 5C). Further, in these genes, RNA Pol II occupancy downstream of the TTS region was increased significantly in the T45A mutant (Figure 5D), demonstrating that the T45A mutation impaired transcriptional termination.

## DISCUSSION

DNA damage induces histone phosphorylation to maintain genomic stability. For example, H2AX-S139 is phosphorylated primarily at DNA double-strand breaks and recruits damage repair complexes (2,3) and H3-T11 is dephosphorylated by Chk1 depletion, suppressing the transcription of cell cycle-related genes (23). Unlike H3-T11 dephosphorylation, which occurs in promoter regions of genes that are repressed on DNA damage, we observed that H3-T45 phosphorylation facilitates the transcriptional activation of DNA damage-inducible genes. Importantly, we demonstrated that AKT phosphorylated H3-T45 in response to DNA damage, which affected transcriptional termination.

**Figure 6.**
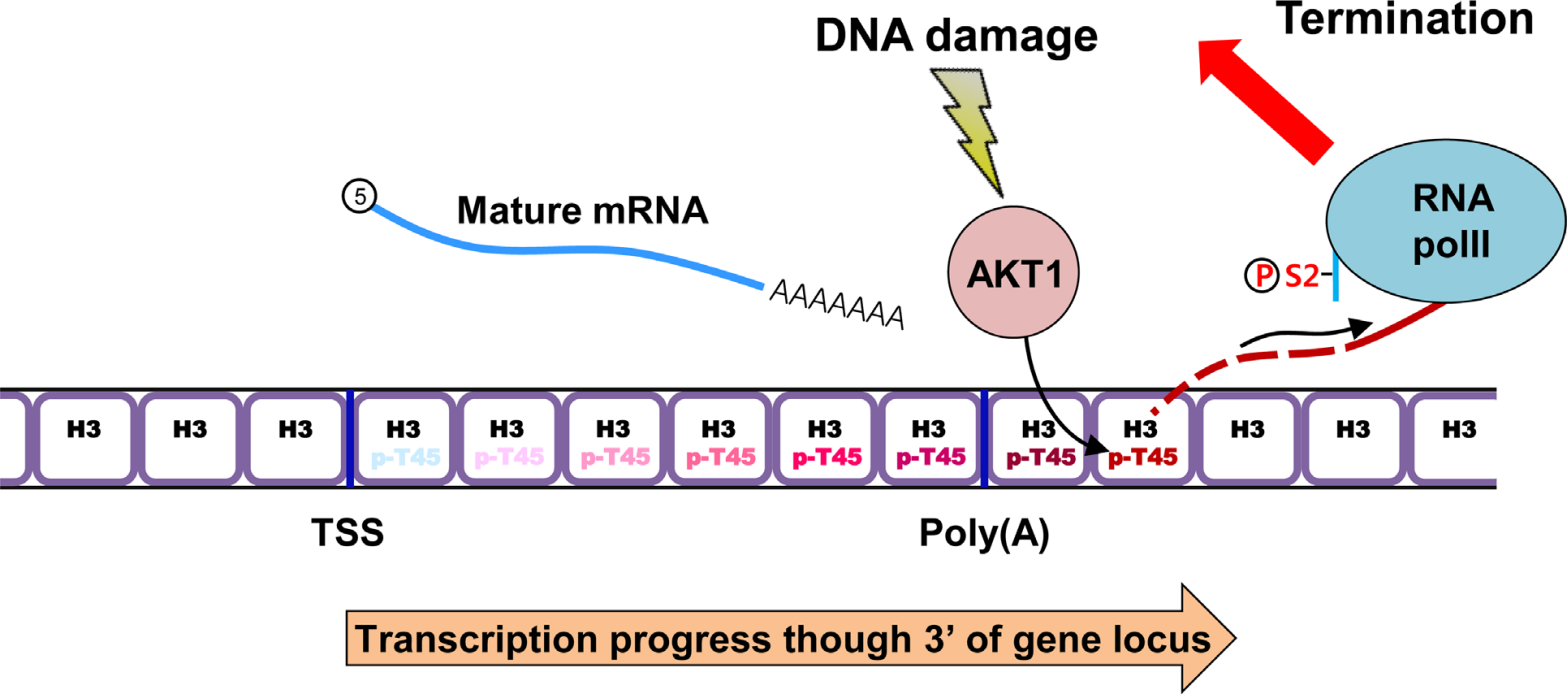
Schematic of AKT1-mediated H3-T45 phosphorylation in transcriptional termination on DNA damage. DNA damage triggers the transcription of stress-response genes by RNA Pol II. As transcription progresses through the 3′ region of the locus, RNA Pol II-S2 undergoes hyperphosphorylation and DNA damage-activated AKT1 phosphorylates H3-T45. When RNA Pol II passes through the 3′ area of the poly(A) site, pre-mRNA is cleaved and is processed into mature mRNA (12,14). Phosphorylated H3-T45 accelerates the degradation of 5′ unprotected RNA downstream of the poly(A) site to induce the dissociation of RNA Pol II from chromatin.

Based on our data, AKT alone is unlikely to differentiate targets for transcriptional termination, because a significant amount of H3-T45 phosphorylation simply follows RNA Pol II-S2 phosphorylation (Figure 3D and E). Also, H3-T45 phosphorylation was not observed in housekeeping genes, in which RNA Pol II-S2 phosphorylation signals were minimal (Figure 3F). It is possible that the factors that correlate with Pol II-S2 phosphorylation (CDK12, for example, is a Pol II-S2 kinase that harbors a conserved AKT phosphorylation motif and is predicted to interact with AKT) (36) recruits AKT to chromatin where transcriptional termination occurs, thereby allowing AKT to phosphorylate H3-T45, facilitating termination (Figure 6).

We wondered whether H3-T45 phosphorylation in other processes, such as DNA replication and apoptosis, correlates with transcriptional termination. Previous studies have suggested that H3-T45 is phosphorylated under different conditions by distinct kinases: PKC-δ under apoptotic conditions (28), Cdc7 during DNA replication (30) and DYRK1A prior to transcriptional activation (32). Except for AKT2, which binds the *CDH1* promoter with Snail1 to repress transcription (37), a link between H3-T45 phosphorylation and transcription has not been reported. Analogous to H3-S10 phosphorylation, which induces transcriptional activation and chromosomal condensation under disparate circumstances (38,39), H3-T45 phosphorylation might function in diverse pathways during various cellular processes. Also, H3-T45 phosphorylation by the kinases above might participate in transcriptional termination.

AKT isoforms are highly homologous but have distinct tissue expression patterns and substrate specificity (40). On DNA damage, AKT1 silencing decreases cell survival, whereas AKT2 silencing has a modest effect (41,42). Thus, AKT1 likely mediates the DNA damage response. Also, we confirmed that AKT1 phosphorylates H3-T45 more effectively than AKT2 (Figure 4), supporting our hypothesis that AKT1 mediates the transcription of stress-response genes through H3-T45 phosphorylation.

Recent studies claim that defective termination at the 3′-end impairs splicing, enhances RNA degradation and reduces transcriptional initiation at the promoter (43), suggesting that overall transcriptional efficiency is governed by transcriptional termination. Thus, the precise mechanism of phosphorylated H3-T45-driven transcriptional termination remains to be elucidated. Discovering the termination factor that recognizes phosphorylated H3-T45 would be paramount to such a study.

In summary, we have provided evidence for the connection between post-translational histone modifications and transcriptional termination for the first time. DNA damage leads to the activation of AKT, which in turn phosphorylates H3-T45, facilitating transcriptional termination for maximum transcriptional efficiency. These observations shed significant new light on the cellular response to DNA damage to regulate transcription through the termination phase.

## SUPPLEMENTARY DATA

Supplementary Data are available at NAR Online.

SUPPLEMENTARY DATA
