# Supplementary material for: AKT phosphorylates H3-threonine 45 to facilitate termination of gene transcription in response to DNA damage
Source: Nucleic Acids Res. 2015 Mar 26;43(9):4505–16. doi: 10.1093/nar/gkv176 (PMC4482061; doi:10.1093/nar/gkv176)
Supplement: SUPPLEMENTARY DATA [file supp_43_9_4505__index.html]

AKT phosphorylates H3-threonine 45 to facilitate termination of gene transcription in response to DNA damage — SUPPLEMENTARY DATA 

# AKT phosphorylates H3-threonine 45 to facilitate termination of gene transcription in response to DNA damage

## SUPPLEMENTARY DATA

**Files in this Data Supplement:**

- SUPPLEMENTARY DATA
- SUPPLEMENTARY DATA
