# Supplementary material for: AKT phosphorylates H3-threonine 45 to facilitate termination of gene transcription in response to DNA damage
Source: Nucleic Acids Res. 2015 Mar 26;43(9):4505–16. doi: 10.1093/nar/gkv176 (PMC4482061; doi:10.1093/nar/gkv176)
Supplement: SUPPLEMENTARY DATA [file supp_gkv176_nar-02623-x-2014-File003.docx]

**Supplementary Data**

**DNA damage-activated AKT phosphorylates H3-threonine 45**

**to facilitate termination of gene transcription**

Jong-Hyuk Lee, Byung-Hee Kang, Hyonchol Jang, Tae Wan Kim, Jinmi Choi, Sojung Kwak, Jungwon Han, Eun-Jung Cho, Hong-Duk Youn

**Supplementary Material and Methods**

Lentiviral sh-RNA-mediated knockdown of CDC7, PKC-δ, and DYRK1A

**Supplementary Figures and Figure Legends:**

Supplementary Figure S1

Identification of histone phosphorylations under DNA damage

Supplementary Figure S2

Comparing H3-T45 phosphorylation and γH2AX upon DNA damage

Supplementary Figure S3

AKT inhibition specifically blocks the phosphorylation of H3-T45 upon DNA damage

Supplementary Figure S4

AKT1 binds and phosphorylates H2B-S36 and H3-T45 *in vitro*

Supplementary Figure S5

Generation of anti-phosphorylated H3-T45 antibody

Supplementary Figure S6

AKT phosphorylates H3-T45 *in vivo*

Supplementary Figure S7

DNA damage induced H3-T45 phosphorylation distributes through DNA damage signaling pathway genes

Supplementary Figure S8

ADR treatment induced H3-T45 phosphorylation on TTS region

Supplementary Figure S9

DNA damage-induced H3-T45 phosphorylation in CDC7, PKC-δ, and DYRK1A knockdown cells

Supplementary Figure S10

H3-T45 phosphorylation is critical for 3’ end processing

**Supplementary Table S1**

Primers used in this study

**Supplementary Table S2**

ChIP profiles of phosphorylated H3-T45, Pol II-S2, S5, and H3K36me3 by adriamycin treatment (provided as a separate file)

**Supplementary Material and Methods**

**Lentiviral sh-RNA-mediated knockdown of CDC7, PKC-δ, and DYRK1A**

Lentiviral vectors containing the human CDC7-targeting sequences pLKO.1-sh-CDC1 #1 (TRCN0000196970), #2 (TRCN0000003171), #3 (TRCN0000196543), and #4 (TRCN0000350364) and PKC-δ pLKO.1-sh-PKC-δ #1 (TRCN0000284800), #2 (TRCN0000379731), #3 (TRCN0000101202), and #4 (TRCN000010193), and DYRK1A pLKO.1-sh-DYRK1A #1 (TRCN0000010611), #2 (TRCN0000010612), #3 (TRCN0000010613), and #4 (TRCN0000010614)were purchased from Sigma. As a control, the pLKO.1 vector was used. Lentivirus was produced according to the manufacturer’s protocol using the BLOCK-iT Lentiviral RNAi expression system (Invitrogen). Twenty-four hours after lentiviral infection, infected cells were selected with puromycin (1 μg/ml) for 2 weeks and then used for experiments. Because pLKO.1-sh-CDC7 #2, pLKO.1-sh-PKC-δ #4, and pLKO.1-sh-DYRK1A #1 were most effective, we used it in the experiments.

**Supplementary Figure S1_ Lee et al**

**
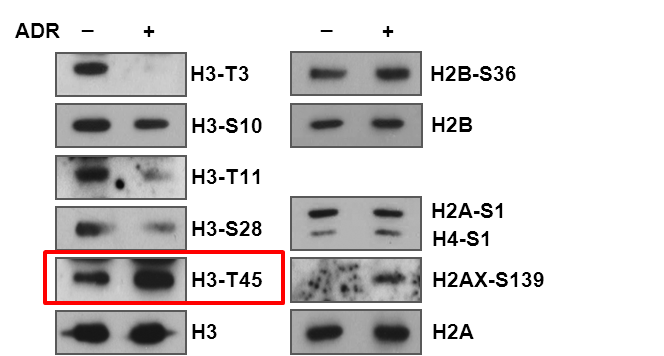
**

**Supplementary Figure S1. Identification of histone phosphorylations under DNA damage.** MCF10A normal breast epithelial cells were treated with DMSO (-) or 0.4 μg/ml ADR for 18 hours. Total cell lysates were probed for western blot with indicated antibodies. Data shown are the representative of three independent experiments.

**Supplementary Figure S2_ Lee et al**

**
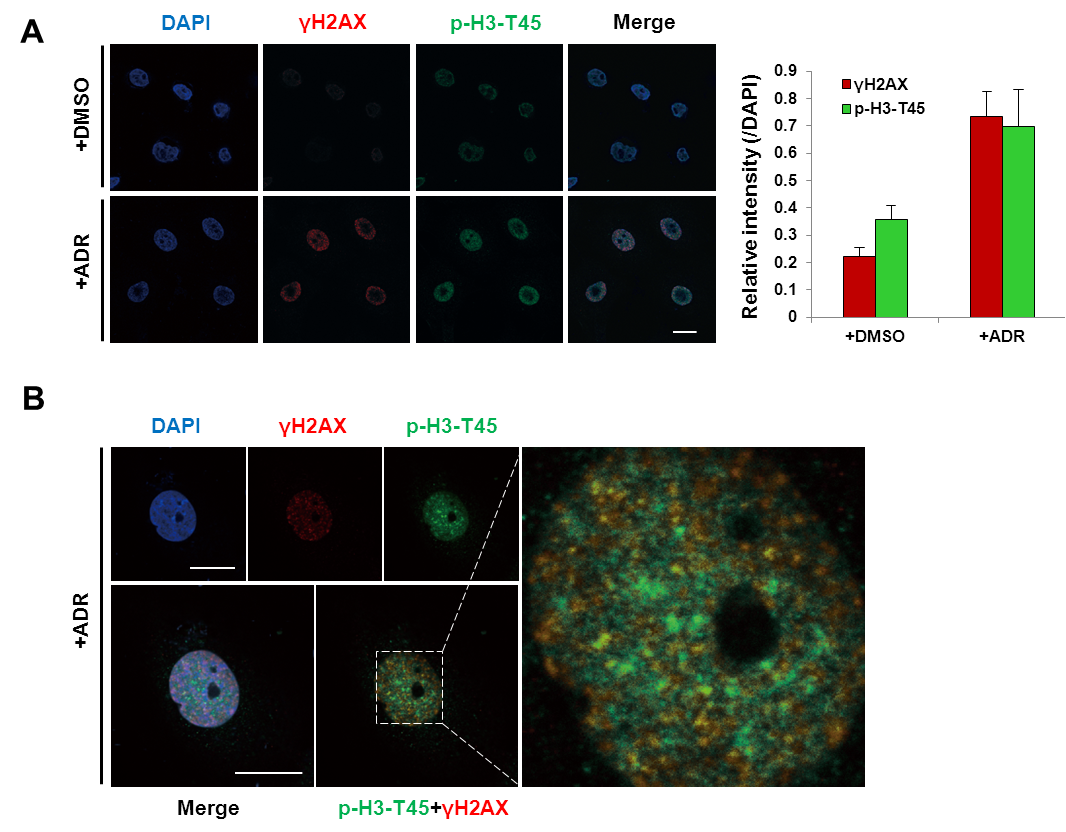
**

**Supplementary Figure S2.** **Comparing H3-T45 phosphorylation and γH2AX upon DNA damage.** (**A**) MCF10A cells, growing on coverslips were treated with 0.4 μg/ml ADR 18 hours. Cells were fixed, permeabilized, stained with anti-phosphorylated H3-T45 (green) and γH2AX (red). DNA counterstained with DAPI. Signal intensity of both signals was compared. Standard deviations are indicated as error bars (n ≥ 3). (**B**) Co-localization of H3-T45 (green) and γH2AX (red), in higher resolution data of a single MCF10A cell in (A). Scale bars 10 μm.

**Supplementary Figure S3_ Lee et al**

**
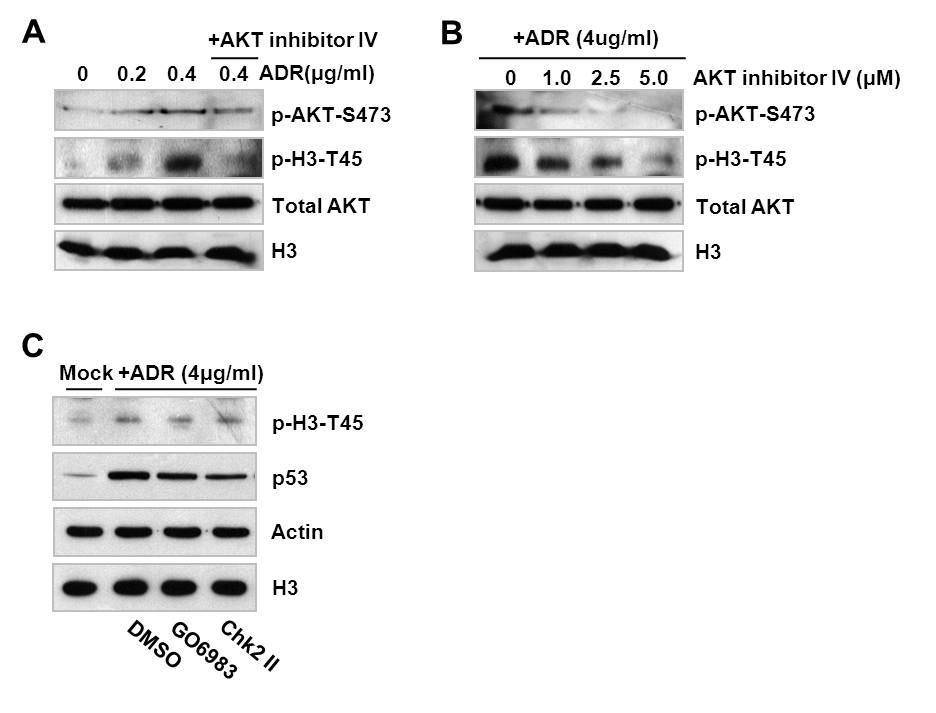
**

**Supplementary Figure S3.** **AKT inhibition specifically blocks the phosphorylation of H3-T45 upon DNA damage.** (**A**) MCF10A cells were treated with 0, 0.2, 0.4 μg/ml of ADR and 0.4 μg/ml ADR + 0.2 μM AKT inhibitor IV for 18 hours. Total cell lysates were probed for western blot. (**B**) MCF10A cells were treated with 0.4 μg/ml ADR with/without indicated concentrations of AKT inhibitor IV for 18 hours. Total cell lysates were probed for western blot. Data shown are the representative of three independent experiments.

**Supplementary Figure S4_ Lee et al**

**
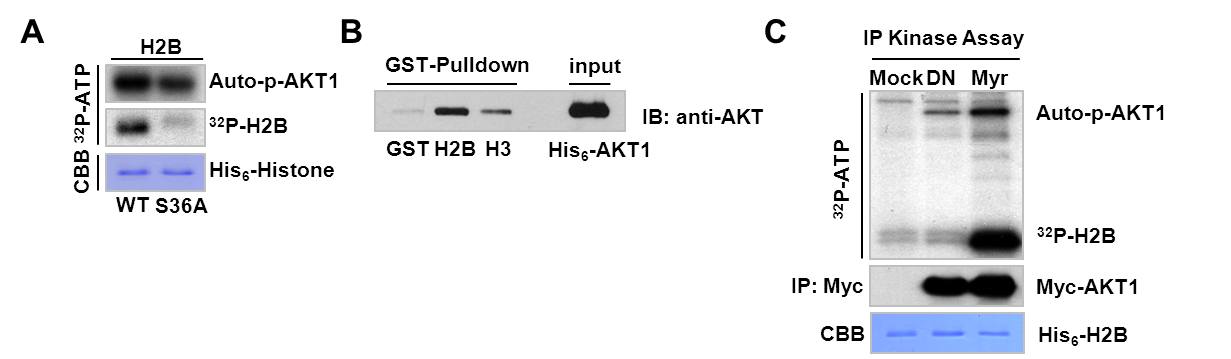
**

**Supplementary Figure S4.** **AKT1 binds and phosphorylates H2B-S36 and H3-T45 *in vitro*** (**A**) AKT1 *in vitro* kinase assay of histone H2B WT and H2B ser36, mutated to alanine (S36A). (**B**) GST-pulldown assay of GST-H2B and H3, mixed with His_6_-AKT1. GST histones were pulled down using glutathione sepharose bead and probed with anti-AKT antibody. (**C**) IP kinase assay of Myc-tagged blank vector, dominant negative (DN), and constitutively-active myristoylated (Myr) AKT1. AKT1 constructs were transfected into HEK293T cells and cell lysates were immunoprecipitated with anti-Myc antibody, subjected to in vitro kinase assay His6-H2B as a substrate. Data shown are the representative of three independent experiments.

**Supplementary Figure S5_ Lee et al**

**
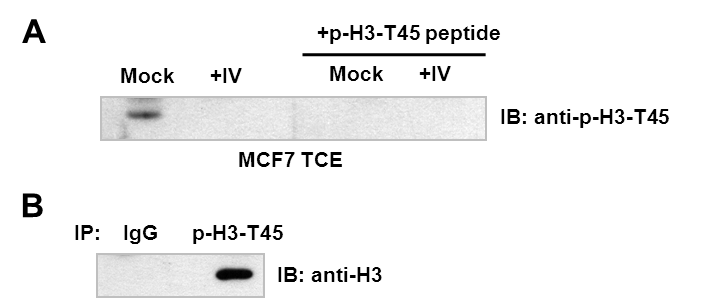
**

**Supplementary Figure S5.** **Generation of anti-phosphorylated H3-T45 antibody** (**A**) Rabbit polyclonal anti-phosphorylated H3-T45 antibody was produced. MCF7 cells were treated with DMSO or AKT inhibitor IV, total cell lysates were probed for western blot. Transferred NC membrane was incubated with anti phosphorylated H3-T45 antibody was blocked with/without phosphorylated H3-T45 peptide. (**B**) MCF7 total cell lysates were immunoprecipitated using normal rabbit IgG or anti phosphorylated H3-T45 antibody and probed for anti H3 antibody. Data shown are the representative of three independent experiments.

**Supplementary Figure S6_ Lee et al**

**
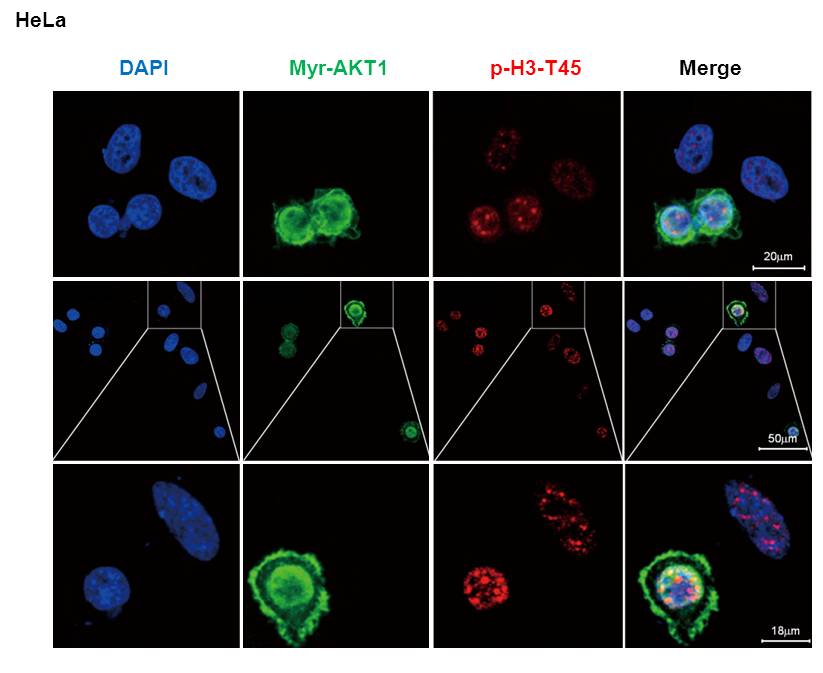
**

**Supplementary Figure S6.** **AKT1 phosphorylates H3-T45 *in vivo*** Immunofluorescence staining for Myc-DN and Myr-AKT1 overexpressed in HeLa cells. DNA counterstained with DAPI. Scale bars are as indicated. Data shown are the representative of three independent experiments.

**Supplementary Figure S7_ Lee et al**

**
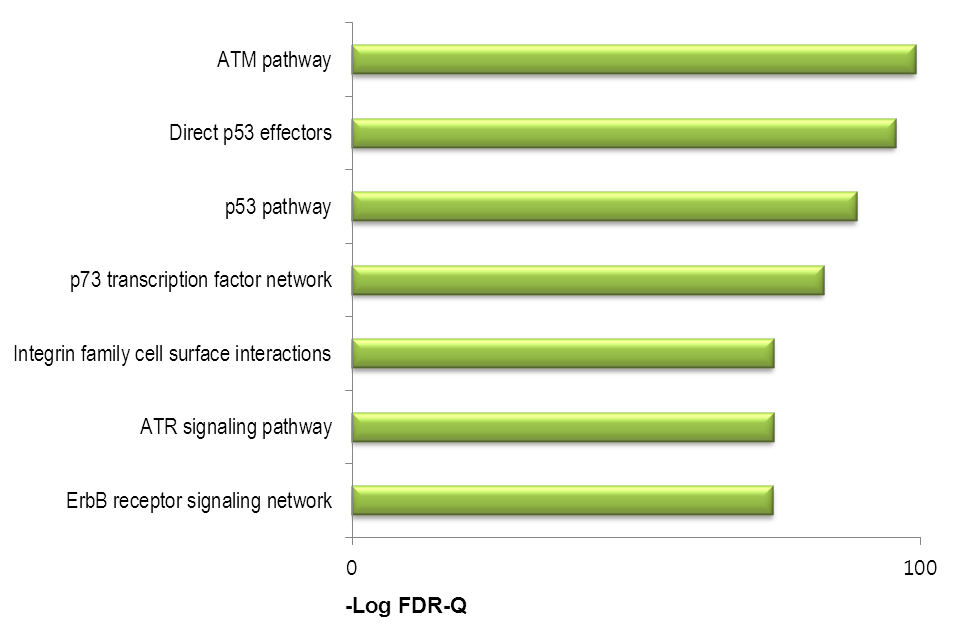
**

**Supplementary Figure S7.** **DNA damage induced H3-T45 phosphorylation distributes through DNA damage signaling pathway genes.** MCF10A cells were treated with 0.4 μg/ml ADR for 18 hours and analyzed by ChIP-seq. Functional annotation (biological pathway) analysis of H3-T45 phosphorylation peak.

**Supplementary Figure S8_ Lee et al**

**
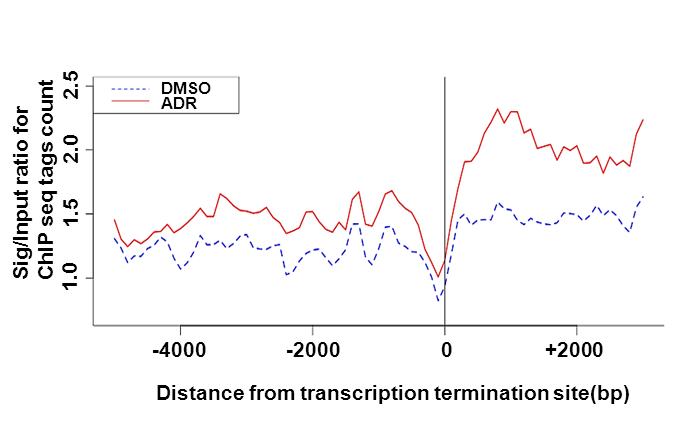
**

**Supplementary Figure S8.** **ADR treatment induced H3-T45 phosphorylation on TTS region.** Average profiles for phosphorylated H3-T45 were plotted around DMSO and ADR induced H3-T45 phosphorylation enriched genes.

**Supplementary Figure S9_ Lee et al**

**
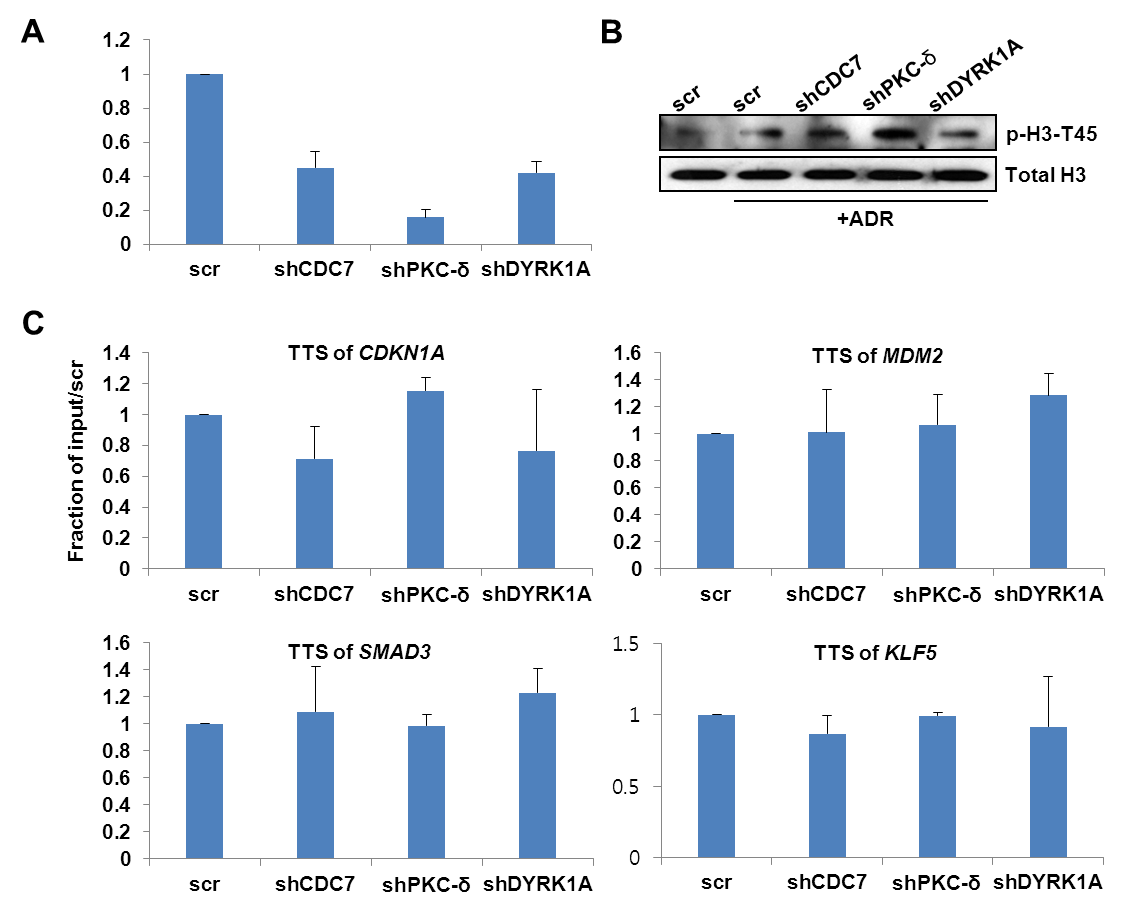
**

**Supplementary Figure S9.** **DNA damage-induced H3-T45 phosphorylation in CDC7, PKC-δ, and DYRK1A knockdown cells. (A)** Real-time qPCR analysis of corresponding mRNAs in lentivirus mediated CDC7, PKC-δ, and DYRK1A knockdown MCF10A cells. **(B)** MCF10A cells were treated with/without 0.4 μg/ml ADR for 18 hours. Whole cell lysates were probed for Western blot with indicated antibodies. **(C)** ChIP assay was performed using anti-phosphorylated H3-T45 in ADR treated MCF10A cells, analyzed with qPCR using primers complement to the TTS of indicated genes. Values were normalized with 1% input DNA/scr. Real-time qPCR and ChIP assay data shown are the average value of at least three independent experiments. Standard deviations are indicated as error bars.

**Supplementary Figure S10_ Lee et al**

**
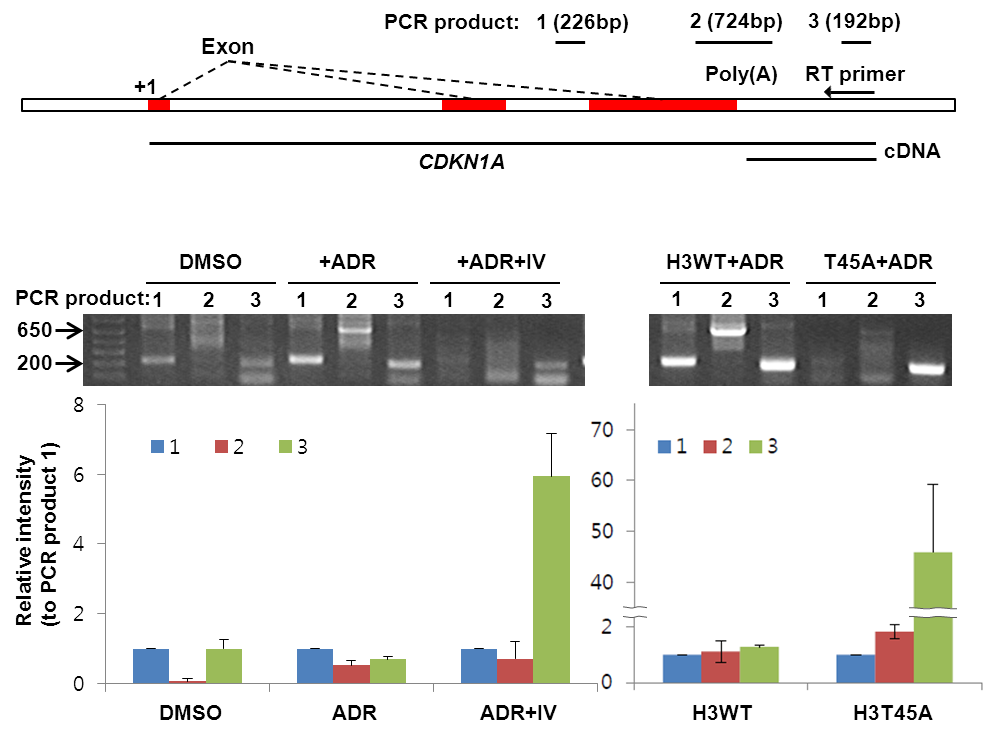
A**

**B**

**Supplementary Figure S10.** **H3-T45 phosphorylation is critical for 3’ end processing. (A)** Schematic of *CDKN1A* locus. (**B**) MCF10A cells and H3 WT or T45A mutant-overexpressing cells were treated with the indicated drugs for 18 hours, and total RNA was reverse-transcribed with reverse-transcription (RT) primer. PCR products were loaded onto 1.1% agarose gels. Blots were quantified using ImageJ (n=3). The values of each group are indicated relative to the intensity of lane 1.

**Supplementary Table S1_ Lee et al**

**Primers used in this study**

| ACTB(Actin) | Forward | 5′-GGCATCCACGAAACTACCTT-3′ |
| --- | --- | --- |
|  | Reverse | 5′-CTGTGTGGACTTGGGAGAGG-3′ |
| CDKN1A(p21) | Forward | 5′-GCAGACCAGCATGACAGATTT-3′ |
|  | Reverse | 5′-GGATTAGGGCTTCCTCTTGGA-3′ |
| AKT1 | Forward | 5′-ATCATGCAGCATCGCTTCTTTGCC-3′ |
|  | Reverse | 5′-ATCTTGGTCAGGTGGTGTGATGGT-3′ |
| AKT2 | Forward | 5′-TCATCAAAGAAGGCTGGCTCCACA-3′ |
|  | Reverse | 5′-TCTCGGTCTTCATCAGCTGGCATT-3′ |
| MDM2 | Forward | 5′-ATGGTGCTGTAACCACCTCACAGA-3′ |
|  | Reverse | 5′-CTTGGCACGCCAAACAAATCTCCT-3′ |
| SMAD3 | Forward | 5′-AGGAGAAATGGTGCGAGAAG-3′ |
|  | Reverse | 5′-CAGTAGATGACATGAGGGAGC-3′ |
| KLF5 | Forward | 5′-CACAAAACATCCAACCTGTCAG-3′ |
|  | Reverse | 5′-CTTGTATGGCTTTTCACCAGTG-3′ |
| CDC7 | Forward | 5′-AGGGATCTGTAGGCCTTTCT-3′ |
|  | Reverse | 5′-GTCTTTGACTGCTTCATGAGTTTC-3′ |
| PKC- δ | Forward | 5′-ACAGCGACAAGAACCTCATC-3′ |
|  | Reverse | 5′-GATCTGTCCAGGAACCTCAATC-3′ |
| DYRK1A | Forward | 5′-CTCGACATCTTCCTCCTCTACT-3′ |
|  | Reverse | 5′-AACGTCCATAGCTCCATTCTG-3′ |
| CDKN1A #1 (ChIP) | Forward | 5′-TGAGGCAGAATTGCTTGAACCTGG-3′ |
|  | Reverse | 5′-TTCACACAGGCTCCCAAGAAGTGA-3′ |
| CDKN1A #2 (ChIP) | Forward | 5′-CTCCCACCCCTACCTGGGCT-3′ |
|  | Reverse | 5′-CAGAACCCAGGCTTGGAGCA-3′ |
| CDKN1A #3 (ChIP) | Forward | 5′-CAGTTCCTTGTGGAGCCGGA-3′ |
|  | Reverse | 5′-TGTGAACGCAGCACACACCC-3′ |
| CDKN1A #4 (ChIP) | Forward | 5′-TGGGGATGTCCGTCAGAACCC-3′ |
|  | Reverse | 5′-CTCCCAGGCGAAGTCACC-3′ |
| CDKN1A #5 (ChIP) | Forward | 5′-TGTCCTTTCCCTTCAGTACCCTCT-3′ |
|  | Reverse | 5′-TAGGGTGCCCTTCTTCTTGTGTGT-3′ |
| CDKN1A #6 (ChIP)  Primer 3 (Fig. S10) | Forward | 5′-CCTCTGCAAAGATCACCAAT-3′ |
|  | Reverse | 5′-CCAGTTGCTCCATAACCTTGCC-3′ |
| CDKN1A #7 (ChIP) | Forward | 5′-TGCACAGGGCAGAGCTTTCTACTA-3′ |
|  | Reverse | 5′-AAACAGGTGCAGGCTATGGGACAA-3′ |
| CDKN1A #8 (ChIP) | Forward | 5′-TGGTTAACATTCAGGCCTTGCTGC-3′ |
|  | Reverse | 5′-ATGGCCACTGTCGATGGAGACATT-3′ |
| RT primer (Fig. S10) | Antisense | CDKN1A #6 (ChIP) Reverse |
| Primer 1 (Fig. S10) | Forward | 5′-GGGTGCGGTGATGGATAAA-3′ |
|  | Reverse | 5′-AGGAGAGACAGCAGAAGTCA-3′ |
| Primer 2 (Fig. S10) | Forward | 5′-AAACGGGAACCAGGACAC-3′ |
|  | Reverse | CDKN1A #6 (ChIP) Reverse |
| MDM2 #1 (ChIP) | Forward | 5′-AGGAAGTTTCCTTTCTGGTAGG-3′ |
|  | Reverse | 5′-TTCTCTGGCCAGTAAGTGATTAG-3′ |
| MDM2 #2 (ChIP) | Forward | 5′-CCGGATTAGTGCGTACGAG-3′ |
|  | Reverse | 5′-TTTCGCGCTTGGAGTCG-3′ |
| MDM2 #3 (ChIP) | Forward | 5′-GGAAATCTCTGAGAAAGCCAAAC-3′ |
|  | Reverse | 5′-CTCAACACATGACTCTCTGGAA-3′ |
| MDM2 #4 (ChIP) | Forward | 5′-AAGTGATCTGCCCGTCTT-3′ |
|  | Reverse | 5′-CCCTGAGAAATAAATGAAATTCACC-3′ |
| MDM2 #5 (ChIP) | Forward | 5′-CCTCCCAAGTAGCTGTGTTATTT-3′ |
|  | Reverse | 5′-GCCTGTAATCCCAGCACTTT-3′ |
| SMAD3 #1 (ChIP) | Forward | 5′-GGGAGGCAGCAAGAGAAAG-3′ |
|  | Reverse | 5′-ACAACAAACCAATTGGCACAT-3′ |
| SMAD3 #2 (ChIP) | Forward | 5′-GTCCATCCTGCCTTTCACTC-3′ |
|  | Reverse | 5′-GAGTTTCTTGACCAGGCTCTT-3′ |
| SMAD3 #3 (ChIP) | Forward | 5′-GCTCCCTCATGTCATCTACTG-3′ |
|  | Reverse | 5′-CAGACCTCGTCCTTCTTCATATT-3′ |
| SMAD3 #4 (ChIP) | Forward | 5′-GACTCTTTCTCAACACAGCTAATTG-3′ |
|  | Reverse | 5′-CTACTCTGGAGGTAAGTGAGGA-3′ |
| SMAD3 #5 (ChIP) | Forward | 5′-ATTAGTGGAAGGCCCAGATTT-3′ |
|  | Reverse | 5′-GGGAGAAAGAGCTATTGGAGAC-3′ |
| KLF5 #1 (ChIP) | Forward | 5′-CTGTCTAATGGTATATTGTGTGTCAAC-3′ |
|  | Reverse | 5′-AGGAAACCACTTCCCTTAATCC-3′ |
| KLF5 #2 (ChIP) | Forward | 5′-AGAGCCTGAGAGCACGGT-3′ |
|  | Reverse | 5′-ACAGCGGCAGGCAGTTT-3′ |
| KLF5 #3 (ChIP) | Forward | 5′-AAATGATCTCTCCTGGTCTGTTC-3′ |
|  | Reverse | 5′-ACCTAAGGAAGCTGATTGCC-3′ |
| KLF5 #4 (ChIP) | Forward | 5′-CAAGTACACCTAGAACCTACATCTT-3′ |
|  | Reverse | 5′-GAAGCAGGACAATCCCTTGA-3′ |
| KLF5 #5 (ChIP) | Forward | 5′-CCTCCCAAAGTACTGGGATTAC-3′ |
|  | Reverse | 5′-GAAGGTGGGTAGATCACTTGAG-3′ |
| CDKN1A  (Nascent RNA RT) | Antisense | 5′-AAACAGGTGCAGGCTATGGGACAA-3′ |
| SMAD3  (Nascent RNA RT) | Antisense | 5′- CTACTCTGGAGGTAAGTGAGGA -3′ |
| KLF5  (Nascent RNA RT) | Antisense | 5′- GAAGCAGGACAATCCCTTGA -3′ |
| CDKN1A #1  (Nascent RNA qPCR) | Forward | 5′-TGTCCTTTCCCTTCAGTACCCTCT-3′ |
|  | Reverse | 5′-TAGGGTGCCCTTCTTCTTGTGTGT-3′ |
| CDKN1A #2  (Nascent RNA qPCR) | Forward | 5′-TGGCAGTAGAGGCTATGGA-3′ |
|  | Reverse | 5′-AAACGGGAACCAGGACAC-3′ |
| CDKN1A #3  (Nascent RNA qPCR) | Forward | 5′-CCTCTGCAAAGATCACCAAT-3′ |
|  | Reverse | 5′-CCAGTTGCTCCATAACCTTGCC-3′ |
| CDKN1A #4  (Nascent RNA qPCR) | Forward | 5′-TCCAGGGTGACAGTGAGATT-3′ |
|  | Reverse | 5′-CTCCCAAAGTGCTGGGATTATAG-3′ |
| CDKN1A #5  (Nascent RNA qPCR) | Forward | 5′-TGCACAGGGCAGAGCTTTCTACTA-3′ |
|  | Reverse | 5′-AAACAGGTGCAGGCTATGGGACAA-3′ |
| MDM2 #1  (Nascent RNA qPCR) | Forward | 5′-GTGTGTAGGTCTGTAGGCTTATG-3′ |
|  | Reverse | 5′-ATGTAATTCAGCATCCACCCA-3′ |
| MDM2 #2  (Nascent RNA qPCR) | Forward | 5′-TGATGGTAACCACAAGTTGTTAATG-3′ |
|  | Reverse | 5′-CTGGTGCTTTCAGATATCTACCTC-3′ |
| MDM2 #3  (Nascent RNA qPCR) | Forward | 5′-CCAACCACACCTGGCTAAT-3′ |
|  | Reverse | 5′-CGCCTGTAATCCCAGTACTTT-3′ |
| MDM2 #4  (Nascent RNA qPCR) | Forward | 5′-AAGTGATCTGCCCGTCTT-3′ |
|  | Reverse | 5′-CCCTGAGAAATAAATGAAATTCACC-3′ |
| SMAD3 #1  (Nascent RNA qPCR) | Forward | 5′-GCTCCCTCATGTCATCTACTG-3′ |
|  | Reverse | 5′-CAGACCTCGTCCTTCTTCATATT-3′ |
| SMAD3 #2  (Nascent RNA qPCR) | Forward | 5′-ACAGGAGATGTAGGGAGAAGAA-3′ |
|  | Reverse | 5′-CTCTAGCCAAGTCACACAGTAAG-3′ |
| SMAD3 #3  (Nascent RNA qPCR) | Forward | 5′-TCAATGGGTGTATCTCGCTATTC-3′ |
|  | Reverse | 5′-GTTGGGTTGGTGTCATTCATTT-3′ |
| SMAD3 #4  (Nascent RNA qPCR) | Forward | 5′-GAGTTTGAGACCAGCCTAGC-3′ |
|  | Reverse | 5′-TTCAAGCGATTCTCCCATCTC-3′ |
| KLF5 #1  (Nascent RNA qPCR) | Forward | 5′-AAATGATCTCTCCTGGTCTGTTC-3′ |
|  | Reverse | 5′-ACCTAAGGAAGCTGATTGCC-3′ |
| KLF5 #2  (Nascent RNA qPCR) | Forward | 5′-CAGAAGAAGAATGGATTGTATGTCAAG-3′ |
|  | Reverse | 5′-AACCCACACATTTGTTCAATGG-3′ |
| KLF5 #3  (Nascent RNA qPCR) | Forward | 5′-AATACACAGTGAGACACAGTAA-3′ |
|  | Reverse | 5′-GTAGCATTTGCTTCCTTAAGTT-3′ |
| KLF5 #4  (Nascent RNA qPCR) | Forward | 5′-GATTATCCTGTCTCAGCATCCC-3′ |
|  | Reverse | 5′-CGTGAATTATGACTCCCTTTATGC-3′ |
